# Impacto de la introducción de un programa externo de categoría 1 en la vigilancia de la estandarización entre laboratorios clínicos en España

**DOI:** 10.1515/almed-2019-0016

**Published:** 2020-01-23

**Authors:** Carmen Ricós, Pilar Fernández-Calle, Fernando Marqués, Joana Minchinela, Ángel Salas, Cecília Martínez-Bru, Beatriz Boned, Rubén Gómez Rioja, Marià Cortés, Elisabet González-Lao, J.V. García Lario, Xavier Tejedor Ganduxé, Sandra Bullich, Montse Ventura, Margarida Simón, Carlos Vilaplana, Ricardo González-Tarancón, Mª Pilar Fernández-Fernández, Francisco Ramón Bauzá, Zoraida Corte, Mª Antonia Llopis, Jorge Díaz-Garzón, Carmen Perich

**Affiliations:** Comité de Programas Externos de la Calidad, SEQC^ML^ , Barcelona, Spain; Comisión de Calidad Analítica, SEQC^ML^ , Barcelona, Spain; Plaza Gala Placidia 2, ático, Barcelona, Spain

**Keywords:** especificaciones de la prestación analítica, estado del arte, estandarización, programas de garantía externa de la calidad, variación biológica

## Abstract

**Introducción:**

El objetivo de este estudio es comprobar la evolución de las especificaciones de la prestación analítica (EPA) utilizadas en programas de garantía externa de la calidad (EQA) y el papel de un programa de categoría 1 en la vigilancia de la estandarización de la prestación de los laboratorios clínicos en España.

**Métodos:**

Se ha revisado la bibliografía sobre tipos de especificaciones de la calidad usados en programas de otros países y se ha comprobado su evolución; se ha comparado el posible impacto de distintas EPA empleadas en ocho países en la toma de decisiones clínicas con tres ejemplos de magnitudes: sodio, tirotropina (TSH) y tiempo de tromboplastina parcial activado (TTPA).

**Resultados:**

Se ha evidenciado la estandarización entre métodos analíticos comprobando si los resultados medios se desvían respecto al valor de referencia certificado del control dentro de las EPA derivadas de la variación biológica (VB). Las EPA usadas en EQA han evolucionado desde el estado del arte hacia la VB. Si se aplican los resultados que se aceptarían con algunas EPA se podrían producir decisiones clínicas erróneas.

**Conclusiónes:**

En España, solo 2 de las 18 magnitudes biológicas estudiadas se pueden considerar bien estandarizadas. Sería necesaria una colaboración más estrecha entre los laboratorios y proveedores de sistemas analíticos para resolver las discrepancias.

## Introducción

El principal objetivo de los laboratorios clínicos es proporcionar información útil que ayude a la toma de decisiones clínicas correctas y, en este contexto, la armonización y estandarización de los procedimientos analíticos es uno de los temas prioritarios hacia donde se dirigen los esfuerzos en la medicina de laboratorio. La armonización permitirá garantizar la comparabilidad de los resultados de magnitudes entre diferentes laboratorios y sistemas sanitarios. Con la estandarización, que es un paso más, se consigue que los resultados de las magnitudes sean trazables a unidades del sistema internacional mediante un método o un material patrón de referencia.

Los resultados obtenidos en diferentes laboratorios deberían ser intercambiables, es decir, estar estandarizados (en el caso de magnitudes biológicas con patrones de calibración y método de referencia disponibles) o armonizados (para magnitudes biológicas sin las condiciones anteriores), para favorecer la toma de decisiones clínicas correctas, independientemente del laboratorio que realice las pruebas analíticas.

La participación en un programa de garantía externa de la calidad (EQA) es un pilar fundamental para la estandarización en la medicina de laboratorio, junto con la colaboración con los fabricantes de productos de diagnóstico *in vitro*, los laboratorios de referencia reconocidos por el *Joint Committee for Traceability in Laboratory Medicine* (JCTLM) [2] y los laboratorios clínicos [1], [3], [4], [5].

El Comité de Calidad Analítica de la IFCC, en 1994, especificó que las comparaciones entre laboratorios deberían diseñarse y llevarse a cabo para asegurar uno o más de los siguientes aspectos [5]: evaluación de los servicios prestados por el participante y de los métodos analíticos utilizados, vigilancia de los dispositivos de diagnóstico *in vitro* y educación continuada, entrenamiento y asistencia.

Un elemento fundamental de un programa de EQA es el razonamiento empleado para definir las especificaciones de la prestación analítica (EPA) y el tipo de EPA utilizado [6], [7]. Existe una relación entre la categoría de la prestación que se pretende conseguir (ideal, excelente, deseable, mínima) y el tipo de EPA utilizado (por ejemplo, límite óptimo derivado de la VB o percentil 20 de los participantes o índice de desviación <1), con independencia del uso clínico que se indique [8].

La capacidad para evaluar la prestación de los laboratorios depende del diseño del programa EQA. Se han descrito cinco categorías (1 a 5, en orden decreciente de capacidad) [9]. Los programas de categoría 1 son aquellos que utilizan materiales de control conmutables con los especímenes humanos, con valores asignados mediante métodos o materiales de referencia, en los que se analizan replicados de los materiales y son los que tienen la capacidad de evaluación más alta.

De acuerdo con todas estas recomendaciones, la Sociedad Española de Medicina de Laboratorio (SEQC^ML^) en 1994 abordó el desarrollo de programas de garantía externa de la calidad (EQA), aplicando un criterio relacionado con el uso clínico de los datos del laboratorio, derivado de la variación biológica (VB), en los informes de evaluación de métodos realizado al final de cada ciclo. Posteriormente, en el año 2001 se incluyó también en la evaluación de los resultados individuales de los participantes. Durante los primeros años todos los programas EQA de la SEQC^ML^ fueron de categoría 4 (controles no conmutables compuestos por especímenes humanos o bovinos, con valores asignados por consenso entre los métodos participantes y análisis replicados de las muestras control) y destinados a monitorizar la prestación de los laboratorios. Desde el año 2015, la SEQC^ML^ ofrece además un programa de categoría 1 destinado a promover la estandarización, que implica obtener resultados equiparables (intercambiables) y trazables a patrones de referencia [10], [11].

El objetivo de este estudio es comprobar la evolución de las especificaciones de la prestación analítica utilizadas en los programas EQA de otros países y el papel de un programa de categoría 1 en la vigilancia de la estandarización de la prestación de los laboratorios clínicos en España.

## Materiales y métodos

Este estudio se ha realizado a partir de los resultados obtenidos en el programa de categoría 1 EQA distribuido en España durante cuatro años (2015–2018).

La metodología aplicada consiste en:

1) Comprobar la evolución de los tipos de EPA usados en programas EQA en los diferentes países, mediante revisión de la bibliografía existente.
2) Comparar el impacto en la toma de decisiones clínicas que podría tener la aplicación de diferentes tipos de EPA en programas de EQA. Para ello se han aplicado las EPA utilizadas en el programa de categoría 1 de la SEQC^ML^, así como las utilizadas en otros ocho programas de diferentes países para tres resultados teóricos de sodio, tirotropina (TSH) y tiempo de tromboplastina parcial activado (TTPA).
3) Evidenciar el grado de estandarización de los resultados de laboratorio en España, evaluando los resultados del programa SEQC^ML^ de categoría 1 durante cuatro ciclos consecutivos (2015 a 2018). Para ello se calcula la desviación porcentual de la media de cada grupo par con respecto al valor de referencia certificado, para cada lote control. Los grupos par están formados por la combinación de método analítico-trazabilidad del calibrador-instrumento; por ejemplo, en la determinación de sodio, un grupo par sería el formado por todos los laboratorios que utilizan electrodo ion selectivo indirecto, trazable al material de referencia NIST-SRM 956, en un analizador automático Abbott Architect.

Se considera que los resultados están estandarizados si las desviaciones porcentuales con respecto al valor de referencia certificado se encuentran dentro de la EPA derivada de la VB para el error sistemático (óptima, deseable o mínima, según la magnitud biológica).

## Resultados

**Figura 1:**
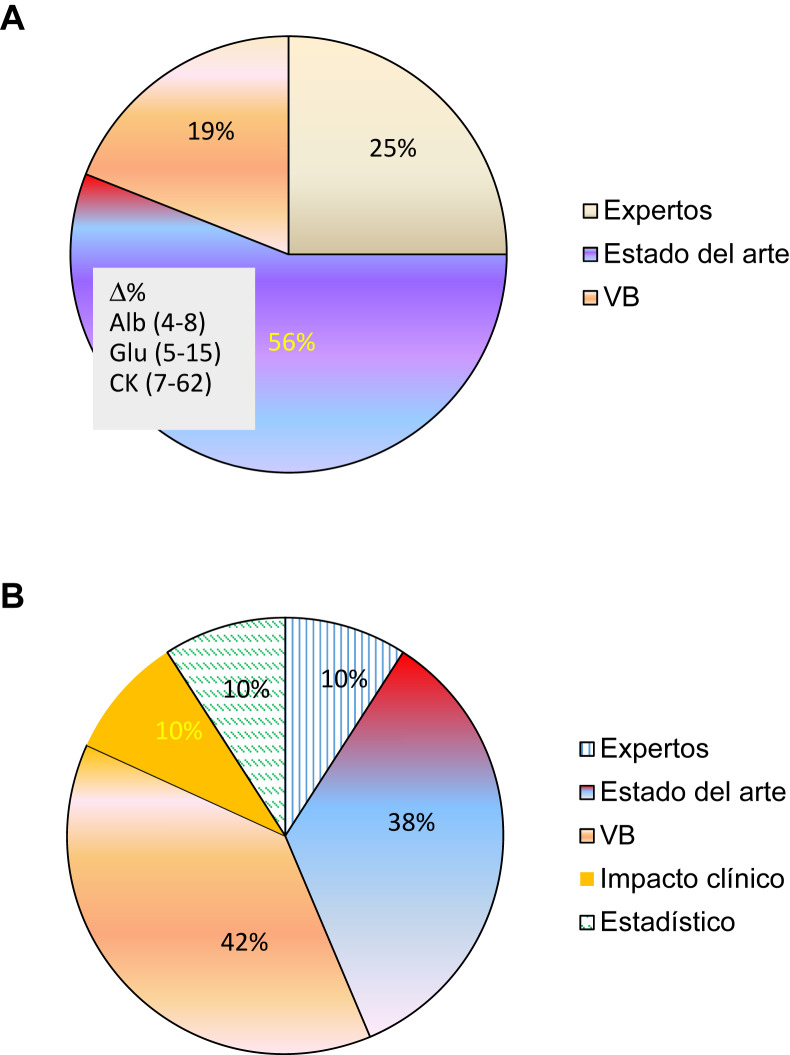
Evolución del tipo de EPA utilizado en programas EQA. (A) 16 programas EQA europeos, 1996. (B) 10 programas EQA mundiales, 2017.

En la Figura 1A, B se muestra la evolución del tipo de EPA usado en programas EQA según evidencias bibliográficas. En 1996 una primera encuesta realizada entre 16 programas EQA europeos puso de manifiesto que la mayor parte de las organizaciones usaban el estado del arte como EPA (prestación existente en aquel momento) (Figura 1A). Este criterio presentaba importantes discrepancias, por ejemplo para la albúmina en suero algunos EQA aceptaban desviaciones respecto al valor diana del 4% mientras otras eran del 8%, para la glucosa desde 5% hasta 15%, para la creatina quinasa (CK) desde 7% hasta 62% [12]. En 2017, 21 años más tarde, una encuesta enviada a organizadores de programas EQA de diferentes países mostró que la mayoría de ellos usaba la especificación en base a la VB [7], tal como se había recomendado en la 1^a^ Reunión Estratégica de la Federación Europea de Medicina de Laboratorio (EFLM) de Milán en el año 2014 [13] (Figura 1B).

Los datos de VB se pueden obtener en las páginas web de la SEQC^ML^ [14], [15] y EFLM [16].

En la Tabla 1 se muestran ejemplos del impacto sobre las decisiones clínicas causado por el tipo de EPA utilizado. En ella se presentan las EPA usadas en los programas EQA de distintos países para algunas magnitudes biológicas. Se puede observar como un paciente con un valor de sodio de 133 mml/L (hiponatremia) podría potencialmente no ser diagnosticado en 6 de los 8 países; una mujer eutiroidea podría ser diagnosticada de hipotiroidismo e innecesariamente tratada en 3 de los 8 países; un trastorno de la coagulación podría no ser detectado en 3 de los 7 países revisados. Hay que matizar, sin embargo, que esta comparación es teórica y no tiene en cuenta el desempeño real de los laboratorios participantes.

**Tabla 1:** Intervalo posible de resultados para un resultado teórico debido al empleo de EPA distintas en programas EQA nacionales. Los valores en negritas indican valores aceptados que podrían dar una falsa indicación clínica.

Magnitud biológica	EPA Resultado	CLIA'19 (USA)	RILIBÄK (Alemania)	UK-NEQAS (Reino Unido)	SKML (Holanda)	NOKLUS (Noruega)	RCPAQAP (Australia)	ASQUALAB (Francia)	SEQC^ML^ (España)
Sodio	EPA	4 mmol/L	3%	2%	0.73%	2%	3 mmol/L (2%)	2.5%	1.1%
	133 mmol/L	129–**137**	130–**137**	130–**136**	132–134	130–**136**	130–**136**	130–**136**	132–134
TSH	EPA	20%	13.5%	12.5%	23.7%	12%	0.6 mU/L (15%)	20%	11.9%
	4.1 UI/L	3.28–**4.92**	3.5–4.7	3.6–4.6	3.1–**5.1**	3.6–4.6	3.5–4.7	3.28–**4.92**	3.6–4.6
TPPA	EPA	15%	10.5%	15%	4.5%	5%	–	20%	6.7%
	1.4	**1.19**–1.61	1.25–1.55	**1.19**–1.61	1.34–1.46	1.34–1.46	–	**1.18**–1.65	1.31–1.49

**Figura 2:**
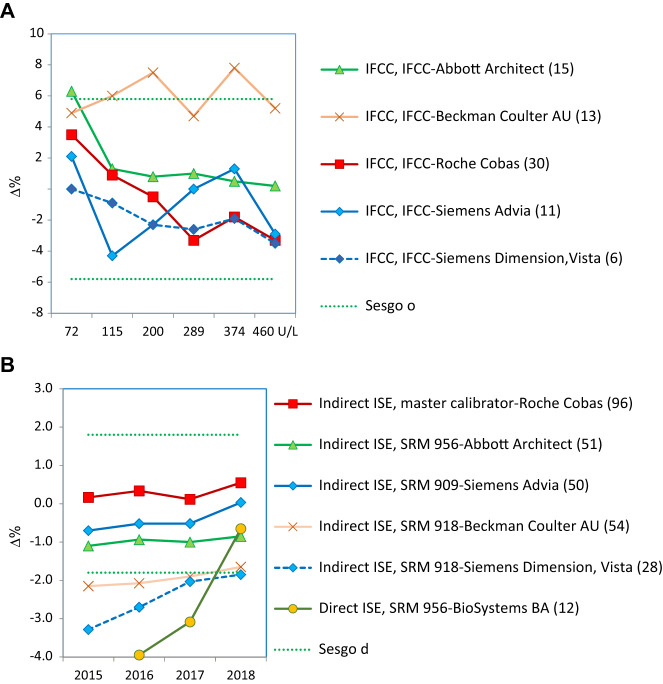
Sesgo obtenido para las magnitudes biológicas estandarizadas. (A) CK (2018). (B) Potasio (2015–2018). Las líneas discontinuas de color verde indican el límite superior e inferior aceptable para el sesgo, derivado de la VB. El límite puede ser el deseable (d) o el óptimo (o). Para cada grupo de comparación se indica entre paréntesis el número de laboratorios participantes.

**Figura 3:**
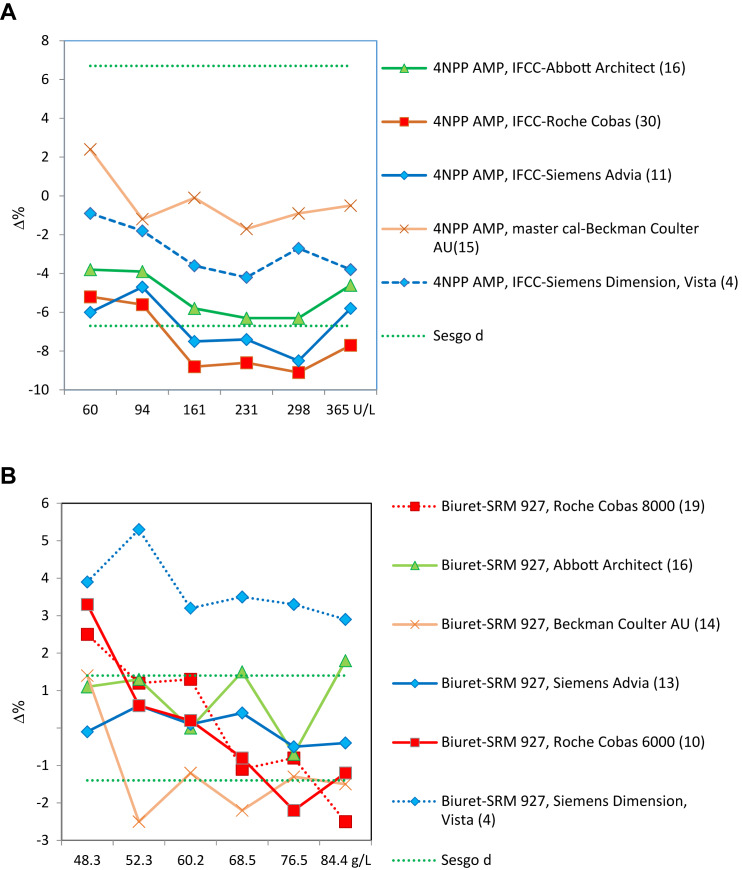
Sesgo obtenido para las magnitudes biológicas no estandarizadas por problemas relacionados con el instrumento utilizado. Las líneas discontinuas de color verde indican el límite superior e inferior aceptable para el sesgo, derivado de la VB. El límite puede ser el deseable (d) o el óptimo (o). Para cada grupo de comparación se indica entre paréntesis el número de laboratorios participantes. (A) Fosfatasa alcalina (2018). (B) Proteína (2018).

**Figura 4:**
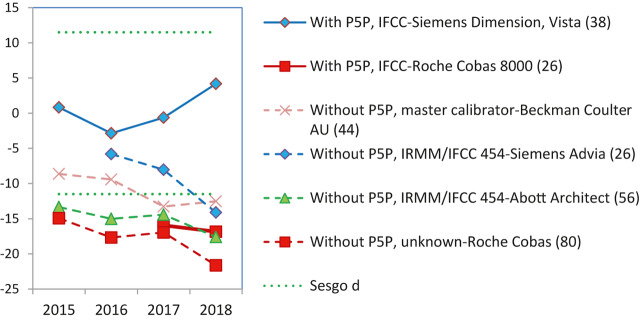
Sesgo dependiente del método utilizado para ALT. Las líneas discontinuas de color verde indican el límite superior e inferior aceptable para el sesgo, derivado de la VB. El límite puede ser el deseable (d) o el óptimo (o). Para cada grupo de comparación se indica entre paréntesis el número de laboratorios participantes.

**Figura 5:**
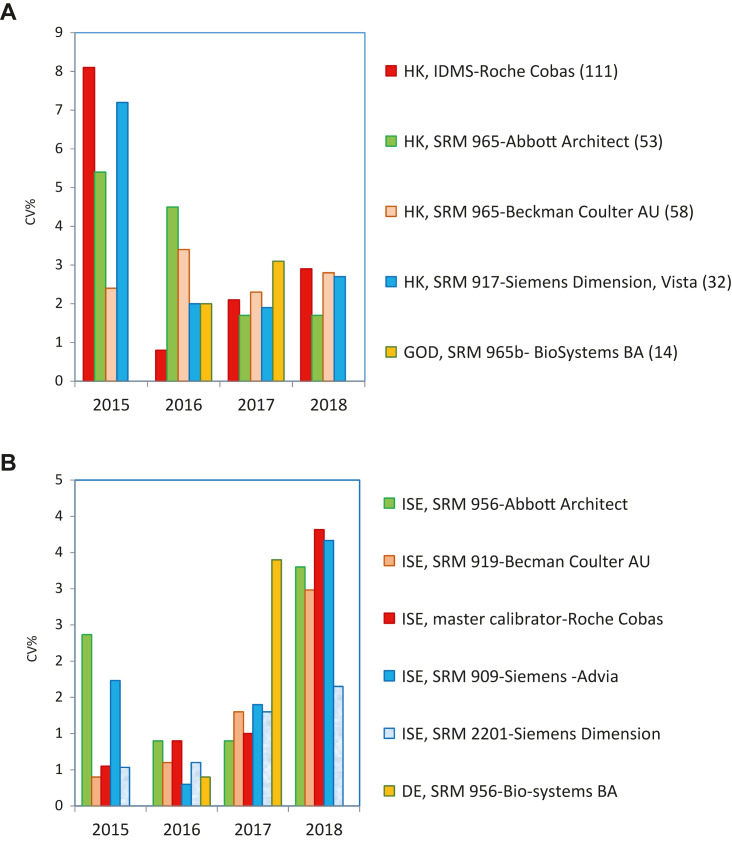
Evolución de la Imprecisión inter-laboratorios (CV%) para glucosa y sodio. (A) Glucosa. (B) Sodio.

Respecto a los resultados de la experiencia de cuatro ciclos del programa EQA de categoría 1 de la SEQC^ML^ (2015 a 2018), se pueden observar cuatro tipos de resultados:

– Sólo se alcanza un adecuado grado de estandarización para CK y potasio (2 de las 18 magnitudes biológicas incluidas en el programa) (Figura 2A, B).
– Se puede ver que para la CK todos los grupos usan el mismo método (recomendado por IFCC, trazable al método IFCC) y todos los instrumentos, excepto Beckman Coulter AU, producen resultados estandarizados (Figura 2A).
– Para el potasio todos los grupos usan el método de electrodo selectivo indirecto (ISE), con distintas trazabilidades. La desviación porcentual de la media general (de los seis controles) obtenida en los cuatro años de experiencia se mantiene dentro del intervalo de la EPA en tres de los seis grupos participantes. Para los otros tres, se puede ver una clara mejora, de manera que prácticamente todos alcanzan la EPA el último año (Figura 2B).
– No se alcanza la estandarización, debido al instrumento utilizado, para fosfatasa alcalina y proteína (Figura 3A, B respectivamente).
– En ambos casos todos los grupos usan el mismo método: el recomendado por la IFCC y trazable al método IFCC para fosfatasa alcalina y el método de Biuret trazable a NIST-SRM 927 para proteína.
– Respecto a la fosfatasa alcalina, dos de los cinco grupos participantes producen resultados mayoritariamente por debajo del límite inferior EPA (6.7%). Se trata de Roche Cobas y Siemens Advia, con desviaciones de −8.3% y −7.8%, respectivamente. Desafortunadamente, son los dos grupos más numerosos en nuestro programa. Braga y cols. [17] observaron la misma desviación negativa para los usuarios de Roche Cobas, en un programa categoría 1 desarrollado en Italia.
– En el caso de la proteína, solo el grupo de usuarios de Siemens Advia obtiene buenos resultados en 2018 para todas las muestras control, mientras que el grupo Siemens Dimension/ Vista produce resultados altos. Los usuarios de los restantes instrumentos dan resultados irregulares en los cuatro años de experiencia.
– No se alcanza la estandarización debido al método empleado para α-amilasa, creatinina, gamma-glutamiltransferasa (GGT), lactato deshidrogenasa (LDH), alaninaaminotransferasa (ALT) y aspartatoaminotransferasa (AST). En la medición de α-amilasa, el método de sustrato maltotriosa produce resultados bajos.
– En la Figura 4 se ilustra, como ejemplo, el caso de la ALT, donde el método que no adiciona piridoxal-5-fosfato (P5P) en el reactivo obtiene una desviación negativa (−18%), independientemente del instrumento-trazabilidad empleado. Idéntica desviación fue observada por Goosens y cols. [18] en un programa de categoría 1 desarrollado en Bélgica. Se debe resaltar que para ALT/AST todos los proveedores en nuestro país ofrecen un falso método “trazable a IFCC” sin la obligación de añadir P5P al reactivo, dejando a criterio del usuario el utilizarlo o no. Esta práctica incorrecta afecta a un 48% de los participantes en el programa y tiene un impacto muy negativo sobre la estandarización.
– En el extremo opuesto se encuentran la creatinina con el método de Jaffe, GGT para el método con sustrato inferior a 4 mmol/L y LDH para el método reverso piruvato a lactato (P–L), que obtienen siempre resultados altos.
– Hay otros siete casos donde no se consigue estandarización que deben ser debatidos con los proveedores, porque producen resultados de imprecisión y sesgo irregulares: bilirrubina, calcio, cloruro, glucosa, magnesio, sodio, urato.
– Por ejemplo, la medición de glucosa muestra una mejora de los coeficientes de variación (CV) inter-laboratorios (promedio anual de los 6 controles) que empezó del orden de 8% para algunos métodos y se ha reducido al 2–3% para todos los métodos, lo cual es una evolución normal y esperada; mientras que el sodio muestra un ilógico ascenso del CV inter-laboratorios con los años (1.0–3.5%) (Figura 5A, B respectivamente).
– Por lo que respecta al sesgo, ninguno o muy pocos grupos participantes obtienen resultados dentro de las EPA en los cuatros años de la experiencia.

## Discusión

La ventaja aportada por la participación en programas EQA de categoría 1 es doble. Por un lado los materiales control conmutables siguen el mismo comportamiento frente a los métodos analíticos que las muestras humanas y, por lo tanto, los indicadores de imprecisión y sesgo (así como la inexactitud de los resultados individuales, no tratados en este estudio), obtenidos pueden ser extrapolados a los resultados de los pacientes.

Por otra parte, al disponer de valores asignados por métodos de referencia certificados, la desviación está calculada con respecto al valor verdadero y, por tanto, se estima el sesgo real.

Una característica adicional es que, con este tipo de programas, la comparación de los resultados entre organizaciones de diferentes programas EQA es válida, porque todos los resultados se evaluaron con respecto a las mismas especificaciones, que son las derivadas de la VB. La preeminencia principal es que estas especificaciones están relacionadas con el uso clínico de las pruebas del laboratorio, son límites fijos e independientes de los sistemas analíticos y, en consecuencia, se pueden compartir con otros laboratorios [19]. Cuando se utilizan los criterios de EPA dispares, se podrían tomar decisiones clínicas equivocadas, como se muestra en la Tabla 1.

Debido al hecho de que nuestros resultados coinciden con otras experiencias EQA, podemos pensar en posibles causas para la no estandarización, como el utilizar distintos procedimientos analíticos para una misma magnitud biológica, como se ha visto en este estudio para creatinina, amilasa sustrato malto-triosa, ALT/AST sin PNP, LDH reversa (P–L). También se han descrito otras causas como la posible variación lote-a-lote de los calibradores, modelo de curva de calibración inadecuada y asignación incorrecta de valores a los calibradores [20].

El uso de EPA derivadas de la VB ha evolucionado favorablemente con los años. Existen ventajas e inconvenientes para el uso de estas especificaciones. El principal inconveniente es que todavía no se conocen datos de VB para todas las magnitudes biológicas analizadas en el laboratorio clínico. La principal ventaja es que la VB está relacionada con el uso clínico de las pruebas del laboratorio para el cuidado de la salud del paciente. Se trata de límites fijos e independientes de los sistemas analíticos; en consecuencia se pueden compartir con otros laboratorios, médicos clínicos y usuarios del sistema de salud.

Los resultados presentados en este artículo conducen a las siguientes recomendaciones:

– El laboratorio clínico debería participar en un programa EQA de categoría 1 para verificar que los resultados obtenidos con un análisis único de las muestras de sus pacientes son exactos, comprobar si el método analítico que utiliza está estandarizado y es trazable a patrones de referencia, así como asegurar que el posible sesgo de sus mediciones es aceptable desde el punto de vista de la VB y, por tanto, compartir intervalos de referencia poblacionales con otros laboratorios que cumplan la misma condición o con los obtenidos de la bibliografía.
– De forma complementaria, puede participar regularmente en un programa EQA de cualquier otra categoría, para monitorizar que su prestación se mantiene con el tiempo y es comparable con la de otros laboratorios usuarios de su mismo método/instrumento, evaluar magnitudes biológicas en los que no existen materiales y métodos de referencia y conocer con qué métodos analíticos y con qué analizadores se obtienen los mejores resultados.
– Todo ello no exime de realizar el control interno de la calidad analítica (diario), que permite aceptar o no las series analíticas y obtener el correspondiente indicador de imprecisión.
– Cuando se utiliza el estado del arte como EPA, el laboratorio debe intentar conseguir compararse con la mejor prestación posible [21], como podría ser el percentil 20 y no con la prestación media (percentil 50) o la más frecuente (percentil 90). Mientras no alcance la mejor prestación, debe enfocar sus objetivos de mejora hacia este propósito.
– Otro aspecto interesante es la utilidad de los programas EQA para mejorar la armonización de los métodos analíticos entre fabricantes y monitorizar los esfuerzos orientados a su consecución. Este artículo, así como las evaluaciones de los programas o las reuniones con los fabricantes, son ejemplos de esta utilidad para el sistema sanitario, además del beneficio directo que aporta al laboratorio.

En resumen, el programa EQA de categoría 1 distribuido en España durante cuatro años demuestra que los resultados en nuestro país todavía no están bien estandarizados para todas las magnitudes biológicas estudiadas. Es necesaria una colaboración más estrecha entre los laboratorios y proveedores de los sistemas analíticos para resolver las discrepancias.

Los organizadores de programas EQA deberían utilizar las especificaciones recomendadas en la conferencia consenso de Milán para asegurar que los resultados de los laboratorios participantes conduzcan a un correcto diagnóstico y seguimiento de los pacientes. Sería conveniente, además, comparar sus resultados con otros EQA para definir correctamente el estado del arte, así como continuar desarrollando programas de categoría 1.
